# Molecular mechanisms underlying sarcopenia in heart failure

**DOI:** 10.20517/jca.2023.40

**Published:** 2023-12-31

**Authors:** Cody A. Rutledge

**Affiliations:** 1 Acute Medicine Section, Division of Medicine, Louis Stokes Cleveland Veteran Affairs Medical Center, Cleveland, OH 44106, USA.; 2 Department of Medicine, Case Western Reserve University School of Medicine, Cleveland, OH 44106, USA.

**Keywords:** Heart failure, sarcopenia, mitochondria, proteostasis, inflammation, skeletal muscle

## Abstract

The loss of skeletal muscle, also known as sarcopenia, is an aging-associated muscle disorder that is disproportionately present in heart failure (HF) patients. HF patients with sarcopenia have poor outcomes compared to the overall HF patient population. The prevalence of sarcopenia in HF is only expected to grow as the global population ages, and novel treatment strategies are needed to improve outcomes in this cohort. Multiple mechanistic pathways have emerged that may explain the increased prevalence of sarcopenia in the HF population, and a better understanding of these pathways may lead to the development of therapies to prevent muscle loss. This review article aims to explore the molecular mechanisms linking sarcopenia and HF, and to discuss treatment strategies aimed at addressing such molecular signals.

## INTRODUCTION

There are approximately 64 million patients suffering from heart failure (HF) globally^[1]^, with 6.7 million cases situated within the United States^[2]^. Age is a major risk factor for the development of cardiovascular disease^[3]^, and the prevalence of HF is known to increase with age^[4]^. Globally, the prevalence of HF continues to grow^[5]^, and the total number of patients living with HF is expected to continue rising as the world population expands and ages^[6]^. Elderly patients, typically defined as those over 65 years old, represent the majority of HF cases, and the proportion of HF patients that are elderly is also expected to grow further in the coming years^[7]^. Age-associated diagnoses, including cachexia, frailty, and sarcopenia, complicate the diagnosis and management of HF, and are all associated with adverse HF-related outcomes^[8,9]^.

Cachexia, frailty, and sarcopenia are all common age-associated clinical diagnoses with similar etiologies, characteristics, and diagnostic criteria^[10–12]^. There are nuanced clinical criteria underlying each of these diagnoses^[9,12]^. Broadly, frailty measures reduced functional capacity, cachexia represents weight loss, and sarcopenia refers to loss of peripheral muscle mass. The European Working Group on Sarcopenia in Older People (EWGSOP) has released guidance for the definition of sarcopenia as follows: patients with (1) low muscle strength have probable sarcopenia (as measured by grip strength or chair stand testing); while (2) low muscle quantity and quality confirms the diagnosis of sarcopenia (as measured by body imaging); and (3) low physical performance qualifies severe sarcopenia (as measured by performance testing; Table 1)^[13]^. This review will focus specifically on sarcopenia, which is associated with a number of adverse outcomes, including disability, poor quality of life, and death^[13]^, and the molecular relationships between sarcopenia and HF.

Pathologically, sarcopenia is characterized by loss of muscle fibers and accumulation of fat within muscle^[14]^, and can be driven by immobility, nutritional deficit, inflammatory diseases, and genetic conditions, among other causes^[15]^. Sarcopenia is associated with multiple adverse outcomes, including impaired daily physical activities^[16]^, increased incidence of falls and fractures^[17]^, hospitalizations^[18]^, and exacerbated mortality risk^[19]^.

Sarcopenia is present between 20%–31% of HF patients^[20,21]^, exceeding estimates of age-related muscle wasting alone^[22]^. Within the heart, myofibers have complex and varied changes during the development and progression of HF, including changes to myofibrillar protein composition and myofibrillar responses to neurohormonal signaling^[23]^. While HF may be associated with myofiber loss in some types of heart disease, such as dilated cardiomyopathy^[24]^, myofibers are not necessarily lost in the heart in all HF patients. Loss of skeletal muscle myofibers, however, is a poor prognostic indicator in HF; its diagnosis is associated with poorer functional class and a higher incidence of cardiac events^[25]^, while muscle wasting is a strong predictor of mortality in HF patients^[26]^. Sarcopenia has a similar prevalence in both HF patients with preserved ejection fraction (HFpEF) and reduced ejection fractions (HFrEF), while mortality in both HFpEF and HFrEF patients with sarcopenia is elevated at 1 year compared to non-sarcopenic patients^[27]^.

**Figure 1.**
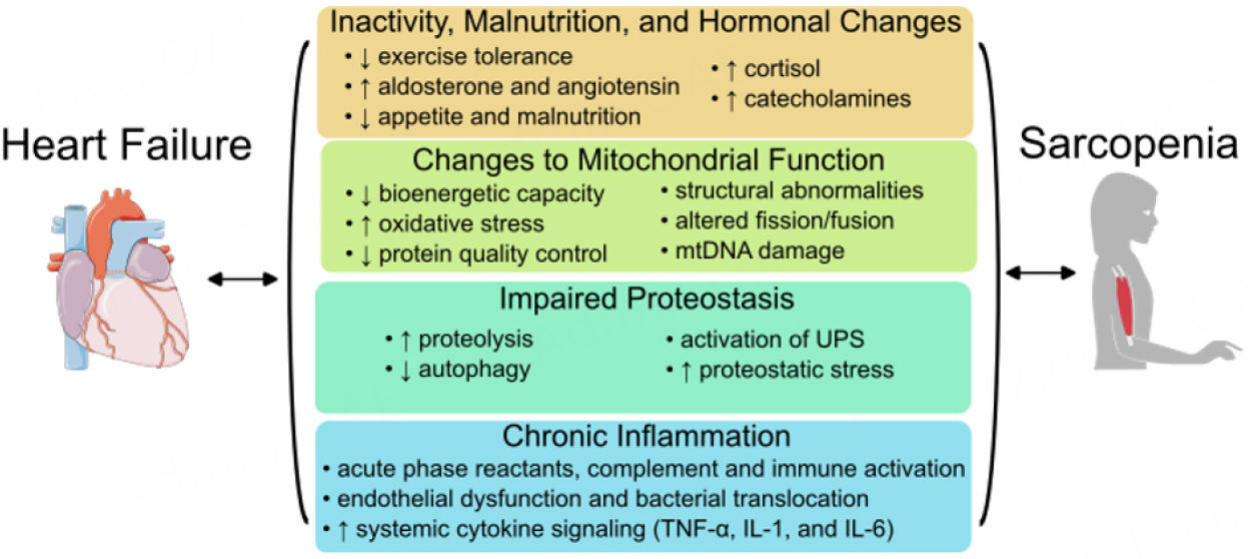
Molecular mechanisms linking heart failure (HF) and sarcopenia. HF patients have reduced exercise tolerance, malnutrition, and altered hormonal signaling, including changes to aldosterone, angiotensin, cortisol, and catecholamine levels, all of which are linked to muscle wasting. Both HF and sarcopenia are associated with mitochondrial changes, including loss of maximal energy production and altered fuel utilization, increased oxidative stress, changes to mitochondrial fission/fusion leading to structural abnormalities, mitochondrial DNA (mtDNA) damage, and reduced mitochondrial protein quality control. Both HF and sarcopenia also have altered protein production and turnover, most notably involving autophagy and the ubiquitin-protease system (UPS), leading to proteostatic stress. Finally, HF is associated with chronic inflammation, particularly as evidenced by increased cytokine signaling involving TNF-α, IL-1, and IL-6, leading to muscle loss.

Despite decades of research, resistance exercise remains the sole therapeutic strategy demonstrated to reliably improve measures of sarcopenia in the elderly population^[28]^. However, exercise tolerance is limited in HF patients, restricting the ability of patients to maintain prescribed exercise. Data on drug therapies designed to improve muscle mass and prevent body mass loss in advanced HF patients is sparse^[29]^. Traditional HF therapies exhibit a degree of beneficial effects in sarcopenic HF patients. For example, treatment with angiotensin-converting enzyme inhibitors (ACEi) was found to reduce the risk of weight loss in HF, though it did not improve measures of muscle mass^[30]^. In order to overcome this treatment deficit, a better understanding of the mechanistic signals underlying shifts in both cardiac and skeletal muscle within sarcopenic HF patients is required to facilitate the development of novel therapeutic strategies. This review will emphasize recent advances in the understanding of mechanisms linking HF and sarcopenia, and discuss potential therapeutic avenues for ameliorating these changes [Figure 1].

### INACTIVITY, MALNUTRITION, AND HORMONAL CHANGES

**Figure 2.**
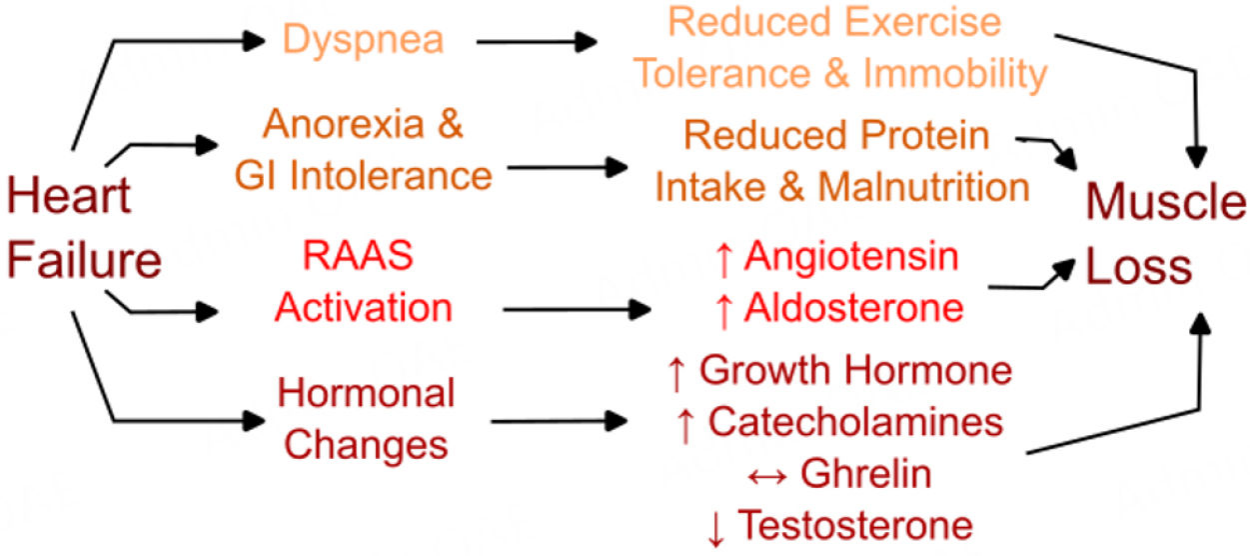
Overview of activity-related, nutritional, and hormonal changes in heart failure (HF) leading to muscle loss. HF patients have exercise intolerance and reduced mobility, leading to reduced exercise capacity. Some HF patients have reduced appetite or digestive issues in the gastrointestinal (GI) tract, leading to malnutrition and reduced protein intake, limiting the ability to sustain muscle growth. HF is also associated with increased renin-angiotensin-aldosterone system (RAAS) signaling, which promotes muscle loss over time. Finally, hormonal changes seen in HF, including growth hormone, catecholamine, ghrelin, and testosterone signaling, all drive an imbalance in catabolism over anabolism, resulting in muscle loss.

Perhaps the most intuitive link between HF and sarcopenia involves physical inactivity in the HF patient. Exercise intolerance and reduced functional capacity are both hallmarks of HF^[31]^ and risk factors for developing sarcopenia^[22]^. Exercise training, particularly resistance exercise, remains the most well-studied and reliable method for improving muscle function in HF patients^[32,33]^, despite the difficulties of implementing exercise training in the HF population^[31]^. However, the hormonal, nutritional, and metabolic changes linking HF and sarcopenia are much more complex than solely attributed to lack of physical activity [Figure 2].

Patients with cachexia in HF are known to have dysregulated neurohormonal signaling compared to non-cachectic patients, including shifts to circulating levels of aldosterone, norepinephrine, epinephrine, cortisol, tumor necrosis factor (TNF), and human growth hormone (GH)^[34]^. Broadly, these shifts are associated with an increase in catabolism and energy expenditure, signaling a mismatch between anabolic and catabolic processes that may underly proteostatic changes in the muscle of HF patients who are losing weight. While targeting such a broad milieu of hormonal shifts is pharmaceutically challenging, one obvious strategy to tip this imbalance towards a healthy compromise is to optimize nutrition in order to meet the increased catabolic needs of the advanced HF patient.

Loss of appetite affects a significant portion of HF patients (10%) and is associated with worse one-year mortality in patients hospitalized with acute, decompensated HF^[35]^. Appetite stimulants, such as megestrol and dronabinol, have been investigated for elderly patients with failure to thrive in the general population, and are typically not recommended^[36]^. Cachectic cancer patients, who have significant overlap in disease presentation and pathophysiology to cachectic heart failure patients^[37]^, are occasionally prescribed appetite stimulants to counteract weight loss. While these drugs have been found to improve body weight in this population, though not necessarily muscle mass, they do not improve mortality^[38]^. There is less evidence available for appetite stimulants in HF patients with weight and muscle loss. While megestrol was found to improve lean muscle mass and cardiac function in a rat model of cachexia-induced cardiomyopathy^[39]^, no human data exists to support its use in HF patients with muscle loss. A wide number of studies have focused on micronutrient supplementation and overall HF outcomes^[40]^, though only a few studies focused on muscle mass as an endpoint. Nutritional interventions, particularly increased protein intake, may offer benefits for HF patients with sarcopenia, though there is no broad clinical data to support improved outcomes related to increased protein consumption^[41]^. However, general best-practice recommendations typically involve a nutrient-rich diet with anti-inflammatory properties, such as polyunsaturated fatty acids, and a Mediterranean-style diet, with hypercaloric or hyperproteic supplements if oral nutrition is not effective^[40]^. Currently, studies involving Mediterranean, hypercaloric, and hyperproteic diets in HF patients with sarcopenia are underway^[42]^. Increased protein and amino acid intake could be simple interventions for patients with muscle loss, and could be of particular importance in HF patients with sarcopenia, though larger-scale human studies are required to support this.

**Figure 3.**
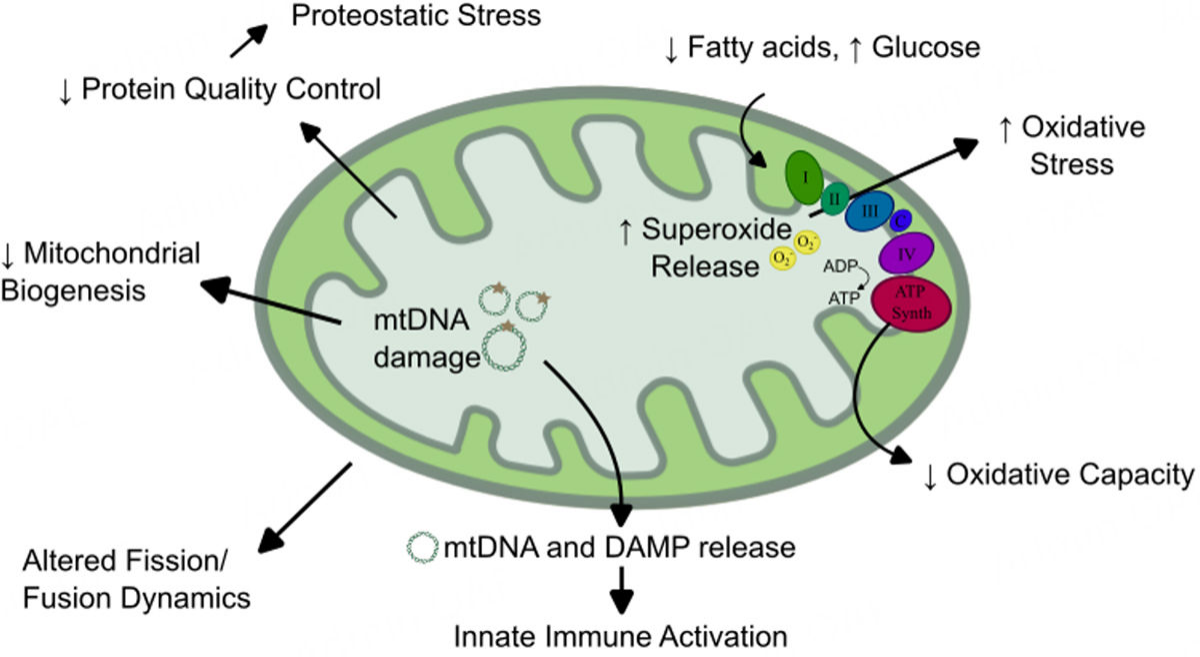
Changes to mitochondrial activity, behavior, and function common to failing myocardium and muscle tissue in sarcopenia. Mitochondria carry out a wide breadth of essential functions in the heart and muscle, and nearly all of these functions are dysregulated in heart failure and sarcopenia. Changes along the electron transport chain (Complexes I-IV and ATP synthase) lead to reduced ATP generation and increase superoxide release. Fuel utilization shifts from predominately fatty acids to alternative fuels, including glucose. Mitochondrial DNAs (mtDNAs) are damaged and released, leading to reduced mitochondrial biogenesis and activation of the innate immune response. Similarly, mitochondrial damage-associated molecular patterns (DAMPs) are exposed, triggering an immune response. Mitochondrial dynamics, including fission and fusion of established mitochondria, are altered, and reduced protein quality control can cause proteostatic stress in the cell.

**Figure 4.**
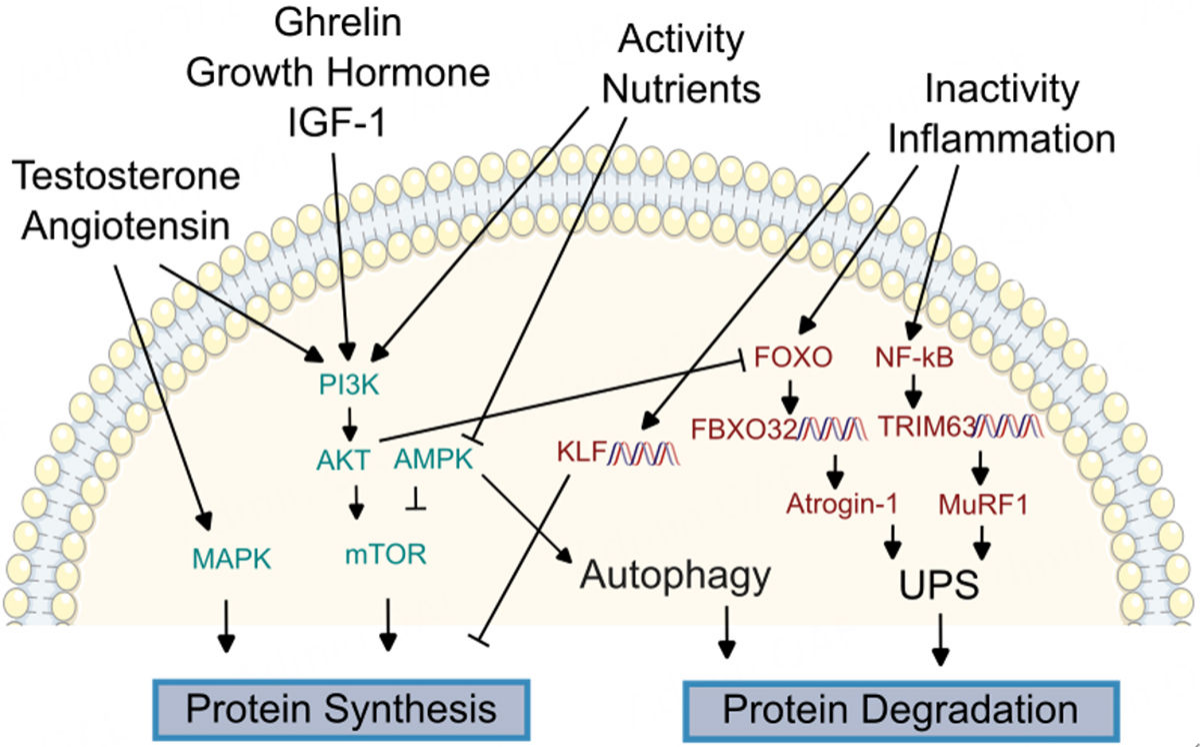
Overview of molecular regulators of proteostasis. Maintenance of the proteome, including synthesis of new proteins and degradation of old and damaged proteins, is dysregulated in both the failing heart and sarcopenic muscle. Signal transducers linking hormonal and nutritional changes to protein synthesis are conserved between the heart and peripheral muscle, including mitogen-activated protein kinase (MAPK), phosphatidylinositol-3-kinase (PI3K) / protein kinase B (AKT), AMP-activated protein kinase, and mammalian target of rapamycin (mTOR). Signals promoting proteolysis include Forkhead box O (FoxO) proteins, which can promote transcription of F-box protein 32 (FBXO32) to increase expression of Atrogin-1 to activate the ubiquitin protease system (UPS). Similarly, activation of nuclear factor κB (NF-κB) triggers transcription of TRIM63 to drive Muscle RING-finger protein-1 (MuRF-1) expression and promote UPS. Kruppel-like factors (KLFs) are promoted by inactivity to inhibit protein synthesis.

Beyond nutritional support, efforts have been made to evaluate specific hormonal targets to improve muscle mass and function in the HF population. Angiotensin signaling, a well-known hormonal change in HF, has been linked to elevated skeletal muscle catabolism through multiple mechanisms, including indirect effects such as vasoconstriction of blood vessels supporting muscle and promotion of mitochondrial dysregulation [Figure 3]^[43]^, as well as direct effects like activation of the ubiquitin-proteasome proteolytic pathway [Figure 4]^[44]^. ACEis are one of the few HF therapies that have demonstrated prevention of weight loss in HF^[30]^. Similarly, aldosterone, a downstream regulator of angiotensin signaling, has been linked to reduced muscle synthesis and increased oxidative stress in muscle cells^[45]^. Aldosterone antagonism may delay muscle loss in the elderly population^[46]^, though HF-specific studies are lacking. More novel hormonal therapies, including targeting of ghrelin and testosterone, are currently being evaluated.

Ghrelin, a peptide generated in the gastrointestinal tract that promotes appetite and alters energy metabolism^[47,48]^, could be a more feasible target than broader appetite stimulants. Circulating ghrelin levels are dysregulated within cachectic HF patients compared to HF patients without cachexia^[49]^. Ghrelin analogs reduce myostatin expression, an inhibitor of muscle growth, in an animal model of HF post-myocardial infarction, providing a direct mechanistic link between ghrelin and muscle loss^[50]^. Among its many other activities, circulating ghrelin promotes cortisol and growth hormone (GH) secretion, and also stimulates insulin-like growth factor (IGF)-1, which promotes protein synthesis via the phosphatidylinositol-3-kinase (PI3K)/protein kinase B (PKB or AKT) pathway (see proteostasis section below for further details; Figure 4)^[51]^. Short-term intravenous administration of ghrelin was found to improve LV function, exercise capacity, and muscle wasting in patients with HF^[52]^, though long-term studies of ghrelin’s outcomes in HF have not been evaluated as yet. However, side-effects of ghrelin supplementation, including sodium and fluid retention that may worsen HF symptoms, lack of oral formulation, and lack of long-term safety data, all limit ghrelin’s mainstream adoption^[53,54]^.

Another promising hormonal target for altering age-related muscle disorders in HF patients is testosterone. Short-term testosterone supplementation has demonstrated improved outcomes in walk-tests in HF patients^[55–57]^, as well as increased muscle mass^[58]^ and improved hand grip^[59]^, albeit with less clear benefits as treatment extended to one year^[60,61]^. While there has been historical concern concerning testosterone supplementation increasing major cardiovascular effects, recent data supports testosterone’s safety in elderly men with hypogonadism^[62]^. The exact mechanism of testosterone on muscle mass remains unclear. *In vitro* studies have found that testosterone increases IGF-1 expression, which may promote protein synthesis via PI3K/AKT signaling, as seen with ghrelin supplementation^[63]^. The increase in protein synthesis has been confirmed in human studies^[64]^. Other *in vitro* studies have linked testosterone supplementation to increased mitotic activity in myoblast cells^[65]^, while another found that testosterone’s effects depend on the mammalian target of rapamycin (mTOR), a protein kinase that plays a major role in cell growth and metabolism^[66]^ [Figure 3]. Regardless of its mechanism, inconsistent clinical benefits and a history of reported adverse cardiovascular events remain major barriers to testosterone’s use for the prevention of sarcopenia in HF.

Recently, novel diabetic therapies have come to market with substantial utilization in patients having cardiovascular disorders^[67]^. Specifically, sodium-glucose cotransporter-2 inhibitors (SGLT2i) and glucagon-like peptide-1 receptor agonists (GLP-1RA) have rapidly increased in popularity within the United States. Their effects on the prevalence of sarcopenia in HF have not yet been fully evaluated, though early reports suggest that both classes of drugs could play a role in muscle loss. SGLT2i drugs inhibit glucose reabsorption within renal proximal tubules to promote urinary glucose excretion. These drugs have recently been recommended for the management of HF^[68]^. SGLT2i were associated with reduced muscle mass in some, though not all, clinical trials in diabetic patients^[69]^. The mechanism of skeletal muscle shifts and cardiovascular benefits of SGLT2i drugs remain unclear, particularly as the primary effect of any SGLT2i appears to occur renally, and a broad number of hypotheses, including fatty acid and ketone metabolism, mitochondrial shifts, nitric oxide availability, and improved endothelial function, are being explored^[70,71]^. However, most current HF studies of SGLT2i are focused on cardiac musculature, rather than peripheral muscle changes. Given the rapid utilization of SGLT2i in HF cohorts, a better understanding of its effects on skeletal muscle in HF patients is warranted.

Similarly, GLP-1RAs, another class of diabetic medications with cardiovascular benefits, may affect skeletal muscle mass and function^[72]^. GLP-1RAs stimulate insulin production and delay gastric emptying, resulting in improved glucose regulation and increased satiety, leading to benefits in the management of both diabetes and obesity^[73]^. Though GLP-1RA’s benefits in HF have been predominantly demonstrated in preclinical models, their mass adoption as weight-loss agents will likely affect HF and sarcopenia outcomes on a population level^[74]^. GLP-1RAs have had mixed reports on muscle mass in animal models, as beneficial effects attributed to anti-inflammatory properties^[75]^ and suppression of myostatin^[76]^ have been identified in aging models. Angiotensin-challenged mice demonstrated reduced muscle mass when treated with a GLP-1RA, which could be related to dysregulated cardiac amino acid metabolism^[72]^. Extensive human studies are warranted to investigate the role of both SGLT2i and GLP1R-a in sarcopenic HF patients, particularly as mass adoption of such therapies is currently underway.

### MITOCHONDRIAL ACTIVITY, BEHAVIOR, AND FUNCTION

Mitochondrial dysfunction is an overly broad term, as mitochondria serve a wide breadth of functions, including, but not limited to, energy production, ion regulation, redox homeostasis, regulation of apoptosis, inflammatory signaling, and steroidogenesis^[77]^. Nearly all of these mitochondrial roles are reduced or dysregulated in the myocardium during HF^[78]^. Mitochondria are established central figures in the pathogenesis of HF, and recent transcriptomic and metabolomic studies in HF models have confirmed their importance^[79]^. Mitochondrial shifts are also heavily implicated in normal aging. Dysregulated mitochondrial fuel metabolism, increased oxidative stress, mitochondrial DNA damage, loss of mitochondrial protein quality control, and altered mitochondrial fission and fusion are all hallmarks of the aging heart^[80]^.

Much like the myocardium, skeletal muscle is highly oxidative and typically contains high mitochondrial density, though mitochondrial content varies significantly by muscle type^[81]^. Skeletal muscle from models of aging-associated muscle disorders demonstrates comparable mitochondrial shifts to those seen in both the aging and failing heart, all of which are generally associated with reduced mitochondrial performance or deleterious mitochondrial signaling to other organelles. These changes include reduced total mitochondrial content^[82]^, mitochondrial structural remodeling^[83]^, altered energetics^[84]^ and reduced maximal bioenergetic capacity^[85]^, increased mitochondrial oxidative stress and dysregulated redox signaling^[86,87]^, reduced mitochondrial protein quality control^[80]^, and increased mitochondria-associated inflammation^[84,88]^. These broad similarities to mitochondrial changes between the failing heart and aging skeletal muscle suggest significant overlap in mitochondrial pathophysiology.

Among the many mitochondrial functions necessary in both the myocardium and skeletal muscle, maintenance of energy through ATP generation has received the most intensive research focus. Altered mitochondrial fuel use has long been known to be a major issue underlying HF, as the failing heart has been described as “an engine out of fuel”^[89]^ and is one of the earlier detectable mitochondrial shifts in the failing heart. Skeletal muscle bioenergetics, including mitochondrial ATP production and substrate utilization, as well as mitochondrial biogenesis, are all decreased in HF patients^[90]^. HF patients also have lower rates of oxidative phosphorylation and longer recovery times in skeletal tissue by nuclear magnetic resonance spectroscopy^[91]^, as well as decreased mitochondrial fatty acid oxidation and increased glucose utilization. Such manifestations are detectable early in cardiac disease, occurring even prior to decreased cardiac contractility^[92]^. Similarly, dysregulated mitochondrial respiration is one of the earliest shifts in skeletal muscle, as demonstrated in HF rodents^[93]^. Skeletal muscle shifts in HF animals follow a similar trajectory to cardiac muscle when measuring mitochondrial respiratory capacity in a HF model^[94]^, again supporting a common mitochondrial phenotypic change between myocardium and skeletal muscle. Though these changes are not commonly measured clinical parameters, advances in respirometry protocols and utilization of circulating platelets as tissue alternatives for evaluating mitochondrial respiratory capacity^[95]^ may eventually lead to clinical testing of bioenergetic capacity with diagnostic and prognostic value.

Peripheral muscle in HF patients also has increased reactive oxygen species (ROS) and mitochondrial structural abnormalities^[96]^, though it is not clear if these are the consequences or drivers of mitochondrial bioenergetic shifts. Mitochondria are the major source of ROS in the heart^[97]^ and a major contributor to ROS generation in peripheral muscle^[98]^. Incomplete reduction of oxygen along the electron transport chain (ETC) can produce superoxide, which can cause broad oxidative damage to the cell, and further mitochondrial damage locally, including damage to mitochondrial DNA (mtDNA).

Mitochondria contain a number of damage-associated molecular patterns (DAMPs) that may trigger the innate immune response^[99]^, as discussed later in this review. In particular, mtDNA, which is highly similar to bacterial DNA, can drive immune activation when released from the mitochondria^[100]^. The most prominent inflammatory cascade related to mtDNA involves activation of the cyclic GMP-AMP synthases (cGAS) pathway, which activates interferon signaling via the Stimulator of Interferon Genes (STING) protein^[101,102]^, a pathway known to increase with aging^[100]^. mtDNAs also lack many of the complex repair enzymes present in nuclear DNA, so they are highly prone to oxidative stress and damage^[103]^. mtDNA damage has been demonstrated extensively in heart disease^[104,105]^ and sarcopenia^[106]^, which in turn lead to ETC dysfunction and reduced mitochondrial biogenesis^[105]^.

The role of mitochondria as either a primary cause or consequence of other signaling pathways common to cardiac and skeletal muscle remains unclear. Systemic signals prevalent in HF, such as increased adrenergic stimulation and renin-angiotensin signaling, are crucial upstream regulators leading to dysregulated mitochondrial bioenergetics in both myocardium and skeletal muscle^[90,107]^, and are already targeted as pillars of HF treatments. Such therapies, including drugs targeting adrenergic and renin-angiotensin cascades such as β-blockers and ACEis, respectively, are known to have myriad cardiac mitochondrial benefits^[108]^. ACEis were found to counter deleterious mitochondrial shifts in the peripheral muscle of rats with HF post-myocardial infarction, leading to improved musculature-based oxidative capacity and normalized expression of mitochondrial transcription factors^[109]^, though the effects of β-adrenergic blockade directly on mitochondria are less clear^[110]^. More broadly, endurance exercise regimens, which remain the strongest recommendation for improving sarcopenia in HF, are known to improve mitochondrial morphology and dynamics in the skeletal muscle of aging patients^[111]^.

Despite the evidence of mitochondrial involvement and broad shifts to mitochondrial functions within both cardiac and skeletal muscle in HF patients, therapies directly targeting the mitochondria remain underdeveloped. Exercise and caloric restriction, which have been shown to protect against mitochondrial disease associated with aging animals, are limited in the HF population^[112,113]^. Mitochondrial ROS scavengers have demonstrated multiple benefits in preclinical models of cardiac disease, though these therapies have largely failed to translate into clinical benefits in human trials^[114]^. Strategies aimed at restoring the balance of nicotinamide adenine dinucleotide (NAD^+^) and its reduced form (NADH), which are coenzymes that are responsible for carrying electrons and crucial regulators of redox metabolism in the mitochondria^[115]^, could be a more viable mitochondrially-targeted therapy. Supplementation with NAD^+^ and treatment with nicotinamide riboside, a precursor to NAD^+^, works to remedy the depleted NAD^+^ pool that has been causally linked to myocardial mitochondria in HF^[116]^. Restoring NAD^+^ levels was also found to be beneficial in models of aging muscle^[117]^. Targeting NAD^+^ could be a promising and easily translatable approach to improving mitochondrial bioenergetic functions in both the myocardium and skeletal muscle of HF patients. An extensive review of mitochondria-targeted therapies for skeletal muscle disorders in HF patients was recently published by Lv *et al*.^[88]^.

Given the breadth of mitochondrial changes seen in failing myocardium and sarcopenic muscle, as well as significant overlap in mitochondrial pathophysiology between these tissues, efforts to ameliorate mitochondrial changes and develop mitochondrially targeted therapies should be promoted in sarcopenic HF models. Work investigating mitochondria in such models should focus on specific mitochondrial activities or functions, be they energetic, signaling, ROS, inflammatory, or proteostatic changes, rather than broadly labeling mitochondrial dysfunction as a driver of disease.

### PROTEOSTASIS

Healthy muscle, in both the heart and peripherally, requires constant synthesis of new proteins together with degradation of old and damaged components, a process known collectively as proteostasis. Proteostasis relies on a number of mechanisms to maintain the proteome, including regulation of protein machinery such as ribosomes, utilization of chaperone proteins to promote protein folding, degradation pathways, such as the ubiquitin-proteasome system (UPS), and autophagy by lysosomes, as well as feedback mechanisms to identify misfolded proteins and protein accumulation^[118]^ [Figure 4]. Dysfunction of proteostasis is a hallmark of aging^[119]^, triggering a stress response in cells. If left unchecked, this dysfunction may eventually overwhelm the cell, leading to cell death or systemic stress signaling via the unfolded protein response^[120]^. Cardiomyocytes are terminally differentiated, so the removal of protein aggregates and damaged protein is critical to avoiding cell death and cardiac fibrosis. Alterations in cardiac proteostasis have been demonstrated within several cardiac disease models, including ischemia^[121]^, arrhythmia^[122]^, and heart failure^[123]^, as well as in the aging heart^[124]^. Alterations in muscle proteostasis are critical changes in the development of sarcopenia as well^[125]^.

There is significant overlap in the molecular regulators of protein anabolism and catabolism between the heart and peripheral muscle. These include highly conserved metabolic sensors, such as PI3K/AKT, AMP-activated protein kinase (AMPK), and mitogen-activated protein kinase (MAPK). As discussed earlier in this review, PI3K/AKT is a central signaling cascade linking external stimuli, such as insulin, GH, or growth factors, to transcriptional regulators of cell growth and protein synthesis^[126]^. Similarly, AMPK is a critical regulator of energy homeostasis and a metabolic sensor^[127]^. AMPK is activated when ATP is depleted, as occurs in cardiac and skeletal muscle tissue in ischemia^[121,128]^, nutrient deprivation^[129]^, and exercise^[130]^. Activated AMPK promotes a wide number of cellular processes, including mitochondrial biogenesis, inflammation, and autophagy, while inhibiting energy intensive processes, such as protein synthesis^[131]^. AKT and AMPK can interact with the activity of each other, and also share a common downstream effector, the mammalian target of rapamycin (mTOR)^[66]^, another protein kinase that plays a major role in cell growth and metabolism^[66]^. Therapies affecting mTOR activity, such as rapamycin, metformin, and resveratrol, are of high interest as anti-aging therapies^[132]^. Finally, MAPK is a highly conserved kinase family able to transduce extracellular changes, including exercise, cytokine signals, and cell stress^[133]^, to regulators of cell proliferation and survival^[134]^. MAPK and AKT have both been identified as downstream regulators for both testosterone^[135]^ and angiotensin^[136]^.

Given the breadth of functions and tissue ubiquity of AKT, AMPK, and MAPK signaling cascades, targeting more specific molecular signals may offer improved insight and therapeutic opportunities towards the prevention of sarcopenia. Regulators of proteolysis may offer such specificity. Aged muscle tissue has increased biomarkers of proteolysis than younger cohorts. While proteasomal activity does not necessarily decrease with age, as measured in muscle biopsies from young versus aged healthy adults^[137,138]^, other biomarkers of proteostatic dysfunction, including impaired autophagy and endoplasmic reticulum stress, have been well-reported in sarcopenia^[125]^. Molecular markers of proteolysis are conserved between heart and muscle in models of skeletal muscle atrophy and heart failure. Such conserved markers include Muscle RING-finger protein-1 (MuRF-1), an E3 ubiquitin ligase that plays a crucial role in protein ubiquitinylation and subsequent degradation^[139]^, and F-box protein 32 (FBXO32, also known as MAFBX or atrogin 1), another E3 ubiquitin ligase.

MuRF-1 is expressed in cardiac and skeletal muscle and is encoded by the gene TRIM63^[140]^. MuRF-1 is upregulated in cell models of skeletal muscle atrophy^[141]^ and in muscle tissues of sarcopenic rats^[142]^. In the heart, MuRF-1 inhibits cardiac hypertrophy in a surgical mouse model of heart failure^[143]^ and has been proposed as a therapy for cardiac hypertrophy in mice^[144]^, suggesting that MuRF-1 has common effects on myofibers across tissues. Overexpression of MuRF-1 can also result in thinning of cardiac muscle and cardiac dysfunction^[145]^. HF can also cause changes to MuRF-1 and proteasomal activity in peripheral muscle. In a rat model of HF, protein expression of MuRF-1 and proteasomal activity is increased in both the diaphragm and quadriceps mucles^[146]^. In humans, MuRF-1 is increased in the skeletal muscle of HF patients, and can be blunted by exercise^[147]^. This may explain, at least in part, the beneficial effects of exercise in HF patients, such as increased protein synthesis and decreased protein degradation that have been demonstrated with exercise in a HF cohort^[148]^. FBXO32 signaling correlates with MuRF-1 in the heart and skeletal muscle, and FBXO32 is highly expressed in muscle tissue during muscle atrophy^[149,150]^, upregulated in animal models of heart failure^[151]^, and down-regulated in cardiac atrophy^[152]^. Similar to MuRF-1, FBXO32 mRNA levels increase in the skeletal muscle of HF rats^[153]^.

The failing myocardium and sarcopenic muscle share transcriptional signals related to proteostasis beyond markers of proteolysis. Two examples are Kruppel-like factors (KLFs), which are DNA-binding proteins that partially control genes for cell growth and differentiation^[154]^, and Forkhead Box O genes (FOXO)^[155]^, which are discussed further in the inflammation section. KLF15 is a major pathway involved in immobilization-induced skeletal muscle atrophy in mice^[156]^ and a wide family of KLF genes have been implicated in heart disease, including HF^[154]^. KLFs have myriad roles, including regulating fuel utilization in both skeletal muscle and the heart^[157,158]^, supporting mitochondrial biogenesis in the heart^[159]^, and mediating vascular inflammatory changes^[160]^.

Autophagy is another important mediator of healthy proteostasis shared between peripheral muscle and the heart. Autophagy is induced by exercise training to promote the healthy turnover of muscle tissue^[161,162]^. However, nutrient deprivation can trigger autophagy through AMPK activation, leading to excessive muscle loss and muscle atrophy^[163]^. Mutations causing excessive autophagy can lead to disproportionate muscle loss in transgenic mice^[164]^. Shifts to autophagy occur early in the progression of muscle loss. Increased markers of autophagy, as well as activation of the UPS, can be detected early within the peripheral muscle of HF rats, prior to evidence of muscle loss that can be detected physiologically^[165]^. Similarly, the upregulation of autophagy-associated genes occurs early after infarction in the peripheral muscle of rats in a myocardial infarction model^[166]^. If unchecked, these shifts may lead to apoptosis in skeletal muscle, which is known to occur in HF patients^[167]^, leading to worsened muscular outcomes. While the search for therapies to manipulate UPS^[168]^ and autophagy in sarcopenia is underway^[169]^, any potential therapies will need to find a healthy balance to maintain muscle, while also promoting alternative pathways of muscle destruction.

### INFLAMMATION

**Figure 5.**
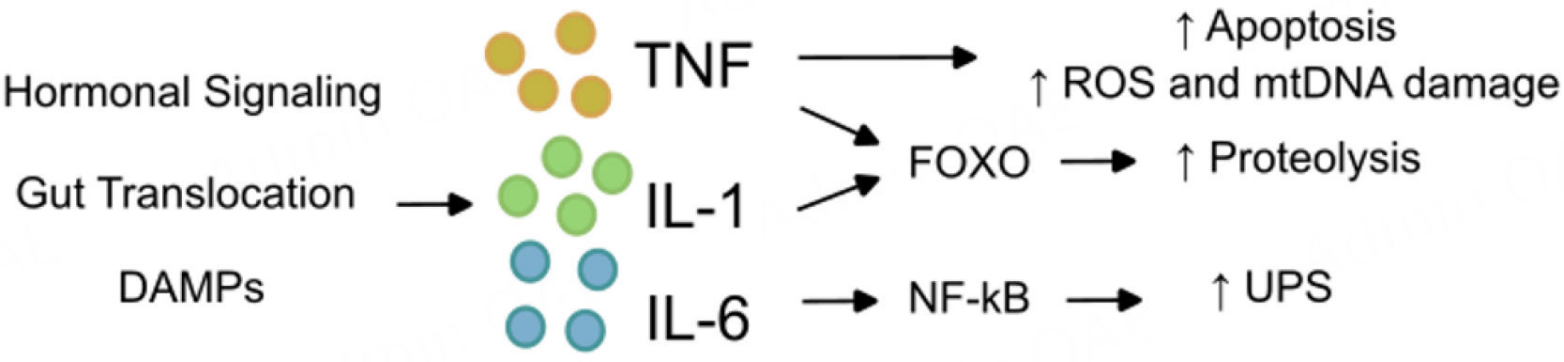
Overview of cytokine changes in myocardium and skeletal muscle during Heart Failure (HF). HF can drive a wide breadth of inflammatory changes. Some of these include elevated pro-inflammatory hormonal signaling, gut edema allowing bacterial translocation, and exposure of damage-associate molecular patterns (DAMPs), leading to the release of cytokines including tumor necrosis factor (TNF) and interleukins (IL) 1 and 6. TNF can cause apoptosis, elevated reactive oxygen species (ROS), and mitochondrial DNA (mtDNA) damage in the musculature, leading to muscle damage and degradation. Transcription factors, including the Forkhead box O (FOXO) nuclear factor κB (NF-κB) families, are activated by cytokines, leading to altered proteostasis and activation of the ubiquitin-protease system (UPS).

Immune activation in HF is widely reported^[170,171]^, with evidence of elevated serum inflammatory markers, specifically tumor-necrosis factor-alpha (TNF-α), in chronic HF patients dating back over 30 years^[172]^. Since that initial observation, a broad number of inflammatory markers, including acute phase reactants, cytokines, and activation of the complement system, have been linked to HF patients^[173]^, both intrinsic and extrinsic to the heart^[170,173]^. Dysregulated circulating cytokine levels can be detected within the blood of HF patients, and several cytokines are predictive of mortality in these cohorts^[174,175]^. Normal human aging has also been linked to dysregulated markers of chronic inflammation, particularly in cardiac stress^[176]^. While the wide breadth of immune activation and various biomarkers of chronic inflammation in HF is beyond the scope of this review, a number of cytokine signals have been linked more directly to muscle wasting in HF patients. Most notably, these include cytokines TNF-α, interleukin (IL)-1, and IL-6 [Figure 5].

TNF-α has been directly linked to the development of HF in animal models. Administration of systemic TNF-α was found to cause the development of LV dysfunction in a rat model^[177]^. Cardiac-specific TNF-α overexpression in transgenic mice not only causes LV dysfunction, but also leads to the disruption of muscle function, including the diaphragm^[178]^. HF patients with elevated levels of circulating TNF have more advanced HF in comparison to HF patients with reduced TNF levels^[172]^. Higher levels of TNF-α, as well as IL-6 receptor levels, are associated with loss of grip strength and muscle mass in an aging population^[179]^, and serum TNF-α levels are a strong predictor of weight loss in HF patients^[34]^.

Mechanistically, Excess TNF-α signaling can promote catabolism through a number of downstream pathways. TNF-α promotes caspase-mediated apoptosis in skeletal muscle, particularly in type II fibers, which may help explain shifts in muscle fiber composition in the aging population^[180]^. TNF-α activation is also linked to dysregulated proteostasis, as TNF-α-treated cachectic rats have reduced protein turnover and increased weight loss compared to untreated controls^[181]^. TNF-α also causes a number of deleterious shifts in cardiac and peripheral muscle tissue, and is known to promote ROS^[182]^ and damage mitochondrial DNA^[183]^.

Similar to TNF-α, the pro-inflammatory cytokines IL-1 and IL-6 are also associated with weight loss, muscle wasting, and frailty in the general population^[184]^, and both have been linked to worsened HF functional class^[185]^. Both IL-1 and Il-6 can trigger activation of nuclear factor κB (NF-κB), a broad family of transcription factors that helps regulate the cell response to infection and can trigger both the innate and adaptive immune response^[186]^ and activation of proteolysis-associated genes^[187]^. NF-κB transcription in muscle cells can lead to activation of the UPS and muscle degradation^[32]^. IL-6 signaling has long been associated with the development of cachexia in cancer patients^[188]^ and, more recently, has been causally linked to muscle wasting in mice^[189]^. In HF patients, systemic IL-6 levels are higher in patient cohorts with muscle wasting compared to HF patients without muscle loss^[21]^. Data on IL-1 association with weight loss in HF patients, however, has been mixed^[21,190]^. IL-1 has a diverse role in functions involving both myocardial contractility and regulation of food intake in the hypothalamus, rendering it a clear candidate to link HF and weight loss^[191]^, although its direct link to muscle loss in HF patients has not yet been proven.

Similar to NF-κB, The Forkhead box O (FoxO) proteins are another family of transcription factors known to be dysregulated in both heart failure^[192]^ and sarcopenia^[155]^. FoxO proteins can be activated by oxidative stress, nutrient deprivation, or DNA damage and regulated by PI3K-Akt signaling^[192]^. More recently, FoxO family signaling has been linked to cytokine signaling in skeletal muscle^[193]^, leading to increased atrogin-1 and promoting muscle catabolism.

While cytokine signaling is one of the best-known links between diseases of aging and muscle loss, in some part due to the ease with which cytokine profiles can be measured in circulating blood, a number of tissue intrinsic pathways also promote immune response. One such pathway involves DAMPS, particularly from the mitochondria. Release of ATP, cardiolipin, and mtDNA from the mitochondria can trigger immune activators including the cGAS-STING pathway, driving pro-inflammatory interferon release^[194]^, or activating the nucleotide oligomerization domain-, leucine-rich repeat-, and pyrin domain-containing 3 protein (NLRP3) inflammasome, which can trigger caspase activity and further drive IL-1 cytokine release^[195]^.

One physiologic mechanism linking HF to immune activation involves congestion of mesenteric veins secondary to fluid overload in the HF patient. This congestion allows for bacterial translocation and endotoxin release from the gut. This concept was originally proposed by Anker *et al.* and has expanded over the last 25 years^[196]^, as has recently been summarized in a review by Liu *et al.*^[197]^. Endothelial cell dysfunction and vessel contractility have long been linked to HF^[198]^, and HF patients with sarcopenia are known to have reduced peripheral blood flow and impaired endothelial cell function compared to HF patients without sarcopenia^[199]^. Reduced peripheral blood flow reduces exercise capacity in HF patients and limits the nutritional support required to sustain peripheral musculature^[200]^. Endothelial cells are major players in the inflammatory process, having varied responses to acute and chronic inflammation that could lead to hyperemia, dysregulated permeability, angiogenesis, and dysregulated blood flow^[201]^. Within the intestinal tract, these alterations could lead to intestinal edema and bacterial translocation, leading to potentially dramatic shifts to systemic inflammation. This mechanism links the fluid overload, common to HF patients, to immune activation via the gastrointestinal tract, and supports a link between diuretic therapy and reduced inflammation in the HF population.

Unfortunately, systemic anti-cytokine therapies have not been found to improve mortality or hospitalization rates in HF patients. Though specific outcomes related to muscle mass have not been reported, treatment with etanercept, a TNF inhibitor, did not improve clinical status in HF patients^[202]^. Similarly, treatment with infliximab, a monoclonal antibody to TNF-α, did not improve HF outcomes^[203]^. Importantly, neither of these focused specifically on biomarkers of sarcopenia. The field of targeted anti-inflammatory therapies is rapidly growing^[204]^, and novel anti-cytokine and anti-inflammatory compounds could offer beneficial effects towards HF-associated sarcopenia in the future.

## CONCLUSIONS

Age-associated muscle disorders, particularly sarcopenia, are associated with advanced HF and lead to poor outcomes and high mortality rates. The prevalence of muscle wasting in HF is expected to grow as HF patients live longer and the global population continues to age. However, the mass adoption of novel drug therapies, including SGLT2is and GLP-1Ras, could shift the prevalence of HF sarcopenia in unpredictable ways. There is a myriad of proposed mechanisms linking HF to peripheral muscle wasting, including limited physical activity and malnutrition in HF patients, abnormal mitochondrial function, dysregulated proteostatic signaling, and systemic inflammation, all of which could be contributing to muscle loss. While traditional HF therapies, including ACEi, β-blockers, and resistance exercise, remain the strongest recommendations for preventing muscle loss, there is promising data supporting testosterone, ghrelin, and mitochondrial-targeted therapies that could gain prominence, though continued human trials and increased knowledge of molecular mechanisms are still critically needed.

## Figures and Tables

**Table 1.** European working group on sarcopenia in older people (EWGSOP2) clinical criteria for sarcopenia

Measurement	Test and criteria according to EWGSOP2^[13]^

Muscle quantity	Grip strength (< 27 kg for men, < 16 kg for women) Chair stand test (> 15 s for five rises)
Muscle quality	Appendicular skeletal muscle mass (ASM; < 20 kg for men, < 15 kg for women) or ASM/height^2^ (< 7 kg/m^2^ for men, < 5.5 kg/m for women) as measured by imaging (dual-energy X-ray absorptiometry, bioelectrical impedance analysis, computed tomography, or magnetic resonance imaging)
Performance	Gait speed ≤ 0.8 m/s, ≤ 8 points on physical battery test, timed up and go test ≥ 20 s, or 400 m walk test ≥ 6 min or noncompletion

## Data Availability

Not applicable.
